# Elective nodal irradiation versus involved-field irradiation for stage II–IV cervical esophageal squamous cell carcinoma patients undergoing definitive concurrent chemoradiotherapy: a retrospective propensity study with 8-year survival outcomes

**DOI:** 10.1186/s13014-023-02332-2

**Published:** 2023-08-28

**Authors:** Jianing Wang, Yajing Wu, Wei Zhang, Yujia Chen, Qing Liu, Shaowu Jing, Jiandong Zhang, Fengpeng Wu, Jun Wang, Xueying Qiao

**Affiliations:** 1 Department of Radiotherapy, The Fourth Hospital of Hebei Medical University, Hebei Clinical Research Center for Radiation Oncology, Shijiazhuang, 050011 China; 2 Department of Radiation Oncology, The First Affiliated Hospital of Shandong First Medical University, Jinan, China

**Keywords:** Cervical esophagus, Squamous cell carcinoma, Elective nodal irradiation, Involved-field irradiation, Survival

## Abstract

**Background:**

Definitive concurrent chemoradiotherapy (dCCRT) is suggested as the standard treatment for cervical esophageal squamous cell carcinoma (CESCC). This retrospective propensity study compared the 8-year survival outcomes and acute treatment toxicities of these patients treated with elective nodal irradiation (ENI) versus involved-field irradiation (IFI).

**Materials and methods:**

Patients with stage II–IV CESCC treated with dCCRT at the Fourth Hospital of Hebei Medical University between January 1, 2007 and December 31, 2020 were enrolled in the study. All the patients were restaged according to the American Joint Commission 8th edition criteria. The propensity score matching (PSM) was used to minimize the effects of treatment selection bias and potential confounding factors including sex, age, ECOG score, clinical T stage, clinical N stage, clinical TNM stage and radiation dose between the ENI group and IFI group. Survival and the prognostic factors were evaluated.

**Results:**

The 131 eligible patients underwent ENI (60 patients, 45.8%) or IFI (71 patients, 54.2%). The median follow-up time was 91.1 months (range, 23.8–182.0 months) for all the patients. The median OS, 1-, 3-, 5-, and 8-year OS rates were 44.4 months, 87.8%, 55.1%, 38.3%, and 27.2%, respectively. After PSM, there were 49 patients in each group. The median OS, 1-, 3-, 5-, and 8-year OS rates for ENI and IFI group were 32.0 months, 83.7%, 48.5%, 38.5% and 31.1% versus 45.2 months, 89.8%, 52.5%, 37.5%, 26.1%, respectively (*P* = 0.966; HR 0.99, 95% CI 0.61–1.61). Similar locoregional control was obtained in both groups. The tendency of leukocytopenia and neutropenia was higher in ENI than in IFI (59.2% vs. 38.8%; *P* = 0.068 and 30.6% vs. 14.3%; *P* = 0.089) at the end of dCCRT.

**Conclusion:**

Cervical esophageal squamous cell carcinoma patients undergoing definitive concurrent chemoradiotherapy has a satisfactory prognosis with organ conservation. The involved-field irradiation might be a better alternative owing to similar overall survival outcomes and local control with less toxicity of myelosuppression.

**Supplementary Information:**

The online version contains supplementary material available at 10.1186/s13014-023-02332-2.

## Introduction

Cervical esophageal cancer (CEC) is an uncommon disease and accounts for only 2–10% of all carcinomas of the esophagus [1]. The most common histology is squamous cell carcinoma in Asia [2]. Cervical esophageal squamous cell carcinoma (CESCC) easily and frequently extends upward to the hypopharynx or downward to the thoracic esophagus. These tumors behave usually very aggressive as they grow in an area of abundant lymphatic drainage and fail to produce early symptoms [3]. Definitive concurrent chemoradiotherapy (dCCRT) is suggested as the standard treatment for CESCC in National Comprehensive Cancer Network (NCCN) guideline. The distribution of lymph node metastases remains unclear because of less opportunity of surgical therapy. Also, due to the low incidence rate, it is difficult to carry out large-scale phase III clinical trials and there were no clearly appropriate irradiation field for CESCC in guideline. Moreover, the target volume definition highly influences the delivery of adequate radiation dose, radiotherapy-related toxicities, and the prognosis for these patients.

Previous studies have reported that three-field lymph node dissection improved local control and long-term survival compared with two-field dissection in operable thoracic esophageal cancer [4], leading to the use of the elective nodal irradiation (ENI) technique in the definitive chemoradiotherapy for thoracic esophageal cancer. Although advanced radiotherapy technology has been widely used for decades, it remains controversial whether the clinical target volume for lymph node (CTVnd) should be adapted with ENI or involved-field irradiation (IFI) [5, 6]. Some oncologists recommended elective irradiation of neck and upper mediastinal lymph node stations to control the potential micrometastasis [7]. However, Ji et al. [8] found that lymph node stations near ESCC received considerable incidental irradiation doses with involved-field irradiation in thoracic esophageal carcinoma. Lyu et al. [9] conducted a prospective, multicenter, randomized, controlled study, concluding that IFI was associated with similar survival as ENI but less acute treatment-related esophagitis and pneumonitis in patients with thoracic ESCC. Despite these, several retrospective studies of cervical esophageal cancer indicated that ENI does not improve survival but increases acute toxicities [10–12]. Moreover, no consensus exists regarding the adequate range of the prophylactic lymphatic area. In our study, we aimed to compare the effects of ENI and IFI on the overall survival (OS) of CESCC patients undergoing dCCRT and the prognostic factors on the OS of these patients.

## Materials and methods

### Patient

The data of patients with cervical esophageal squamous cell carcinoma who were treated by chemoradiotherapy with ENI or IFI were retrospectively reviewed in the Fourth Hospital of Hebei Medical University. Initial staging consisted of medical examination, endoscopy, esophageal barium radiography, computed tomography (CT) of neck, chest and abdomen, and 18 F-fluorodeoxyglucose-positron emission tomography (18 F-FDG PET) if possible which was not covered by medical insurance. All the patients were restaged according to the American Joint Commission (AJCC) 8th edition criteria. Patients were considered eligible for this study if they met the following criteria: (1) histologically confirmed esophageal squamous cell carcinoma; (2) primary tumor located in cervical esophagus; (3) being treated with definitive radiotherapy using three-dimensional conformal radiotherapy (3D-CRT) or intensity-modulated radiotherapy (IMRT) and concurrent chemotherapy. Ineligible criteria are as follows: (1) non-squamous cell histology or multiple primary cancers; (2) distant metastases; (3) Eastern Cooperative Oncology Group (ECOG) performance status (PS) score > 2; (4) history of malignant neoplasm; (5) severe dysfunction of organs such as heart, liver and kidney and so on; (6) patients who did not receiving dCCRT; (7) radiotherapy dose < 50 Gy and (8) missing clinical data. The retrospective collection and analysis of data were approved by the Fourth Hospital of Hebei Medical University Review Board.

### Radiotherapy

Radiotherapy was given concurrently with chemotherapy and delivered by linear accelerators of 6MV X-rays.

The gross tumor volume (GTV) included the primary tumor (GTV-t) and involved regional lymph nodes (GTV-nd) according to endoscopy, barium meal, CT and 18 F-FDG PET if possible.

The clinical target volume (CTV) included the CTV-t, CTV-nd. CTV-t was defined as the primary tumor plus a 2- to 3-cm expansion superiorly and inferiorly along the length of the esophagus and a 0.5 to 0.8 cm radial expansion.

The CTV-nd in IFI group included the GTV-nd and 0.3–0.5 cm margin with or without the involved nodal regions and adjusted appropriately according to the anatomical barrier. For example, there is a patient (T3N1M0) with unilateral supraclavicular lymph node (+). IFI CTV-nd only includes the area of unilateral supraclavicular lymph nodes.

In the ENI group, the CTV-nd covered the elective nodal regions and the lymph node regions where the metastasis located, including the lower neck, bilateral supraclavicular fossa, and upper mediastinum (from the cricoid cartilage to the lower border of the azygos vein). If hypopharyngeal invasion was present, the upper border extended to the hyoid.

The planning target volume (PTV) included the PTV-t and PTV-nd. Expansion of 0.5 to 0.8 cm around CTV-t was defined as the PTV-t. The PTV-nd was generated by applying a 0.3 cm margin to the CTV-nd.

The prescribed dose was 50–66 Gy for 95% PTV in 25–33 fractions (1.8–2 Gy per fraction) with one time a day and five days per week.

The dose limitation to OARs was mean lung dose < 15 Gy and V20 < 25% for lung; V30 < 40% and V40 < 30% for heart; and a maximum dose of < 45 Gy for spinal cord.

### Chemotherapy

Chemotherapy was administered concurrently with radiotherapy. The regimens of chemotherapy included FP (cisplatin and 5-fluorouracil) or TP (paclitaxel and cisplatin) or S-1. Consolidation chemotherapy was allowed to conduct between 4 and 6 weeks after the completion of dCCRT.

### Patient follow-up

The baseline evaluation included physical examination, evaluation of ECOG PS, complete blood cell counts (CBC), serum chemistries, pulmonary function tests and enhanced cervical/thoracic/abdominal CT. CBC was done weekly and serum chemistry was tested every 3 weeks during the dCCRT period. All patients were followed up 6 weeks after the completion of dCCRT, then every 3 months up to 2 years and 6 months to 5 years, and annually after 5 years. The regular follow-up evaluations after the completion of dCCRT included enhanced cervical/thoracic/abdominal CT and barium swallow radiography. Endoscopic examination and biopsy were undertaken if the patient showed signs of recurrence. Suspected neck and supraclavicular node recurrences were confirmed by fine needle aspiration biopsy. Acute radiation injuries were graded according to the Radiation Therapy Oncology Group criteria. Acute hematologic toxicity and gastrointestinal toxicity were graded according to the Common Terminology Criteria for Adverse Events (CTCAE) version 5.0.

### Statistical analysis

Survival time was defined the time from the confirmation of diagnosis to the time of death or follow-up deadline. SPSS version 26.0 software was used for all statistical analyses. The Chi-square test was used to compare the baseline characteristics between the two groups. The Kaplan–Meier method was used to estimate overall survival (OS), and log-rank test was used to ascertain significance between the two groups. The COX proportional hazards model was used to determine the prognostic factors for survival. Variables with *P* value < 0.1 in univariate analyses were included in multivariate analyses. To minimize the effects of treatment selection bias and potential confounding factors between the groups, the propensity score matching (PSM) was conducted with sex, age, ECOG score, clinical T stage (cT), clinical N stage (cN), clinical TNM stage (cTNM) and radiation dose in the covariates at a ratio of 1:1 for ENI group versus IFI group. All *P* values are two-sided and *P* values of less than 0.05 were considered to be statistically significant.

## Results

### Patient characteristics

**Fig. 1:**
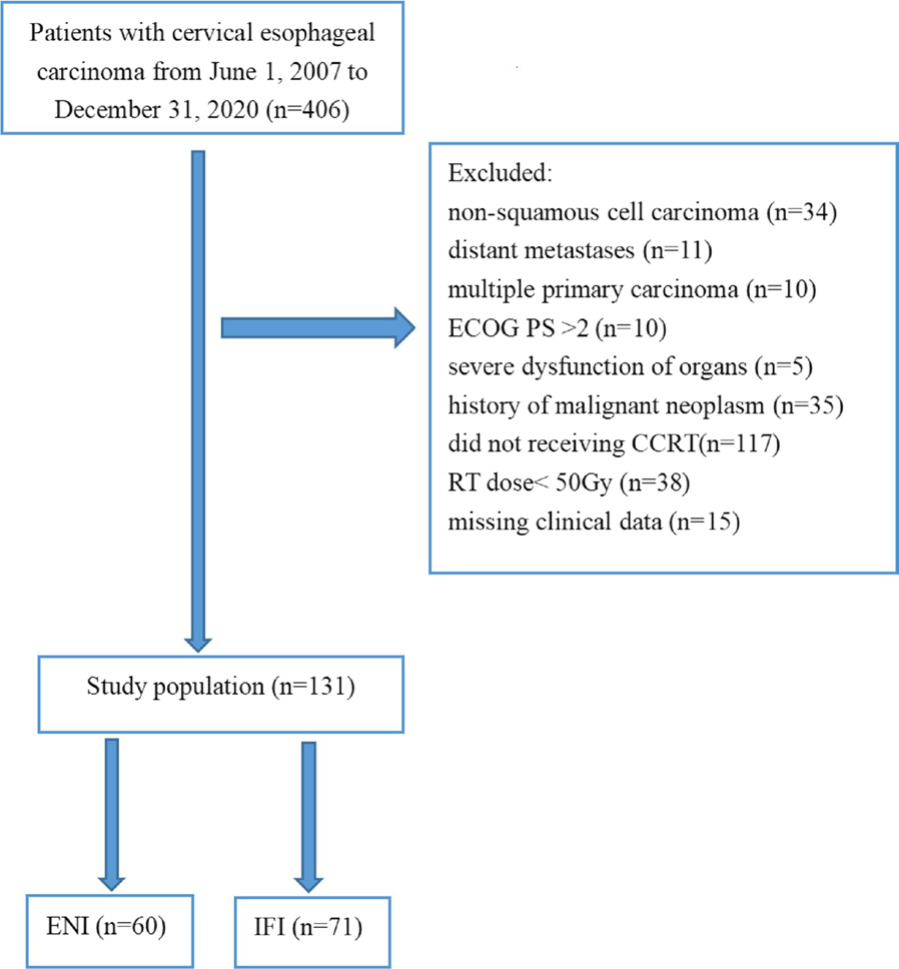
Flow of study enrollment

There were 406 patients with CESCC during the study period. After assessment of eligibility, a total of 131 patients with CESCC were included in the study with 60 patients (45.8%) in the ENI group and 71 patients (54.2%) in the IFI group. The study enrollment was demonstrated in Fig. 1. After PSM, there were 49 patients in each group. The eligible patients’ baseline characteristics were listed in Table 1. Before PSM, there were more patients with the age of > 65 years in IFI group than in ENI group. The median age at diagnosis was 62 (42–74) years in the ENI group and 65 (38–75) years in the IFI group before PSM. The median radiation dose was 60 Gy (range, 50.4–66 Gy) and 60 Gy (range, 50–66 Gy) respectively for ENI group and IFI group. One hundred and twenty patients (91.6%) were treated with IMRT, 11 patients (8.4%) were treated with 3D-CRT. Most of the patients received FP or TP regimen. Seventy-eight patients (79.6%) received 2 cycles of concurrent chemotherapy and 20 patients received 1 cycle of concurrent chemotherapy. Nine patients in ENI group and 6 patients in IFI group underwent induction chemotherapy of 2 cycles. Twenty-eight patients and 31 patients respectively in ENI group and IFI group received consolidation chemotherapy of 1–4 cycles (median: 2 cycles).

**Table 1:** Patient and tumor characteristics

Characteristics	Before matching (n = 131)			After matching (n = 98)		
	ENI (n = 60)	IFI (n = 71)	*P*	ENI (n = 49)	IFI (n = 49)	*P*
Sex			0.602			0.842
Male	30	39		26	25	
Female	30	32		23	24	
Age (year)			0.044			0.520
≤ 65	45	41		35	32	
> 65	15	30		14	17	
ECOG PS			0.438			0.543
0	54	60		44	42	
1–2	6	11		5	7	
cT stage			0.340			1.000
3	48	62		41	41	
4	12	9		8	8	
cN stage			0.511			0.795
0	13	22		12	11	
1	27	29		24	24	
2–3	20	20		13	14	
8th AJCC stage			0.482			1.000
II–III	48	61		41	41	
IV	12	10		8	8	
Radiation dose			0.082			1.000
≥ 50 Gy, ≤ 59.4 Gy	21	15		15	15	
> 59.4 Gy, ≤ 66 Gy	39	56		34	34	
Hypopharyngeal invasion			0.815			1.000
Yes	9	12		7	8	
No	51	59		42	41	

*ECOG* Eastern Cooperative Oncology Group; *PS* Performance status; *AJCC* American Joint Commission

### Survival and prognostic factors

**Fig. 2:**
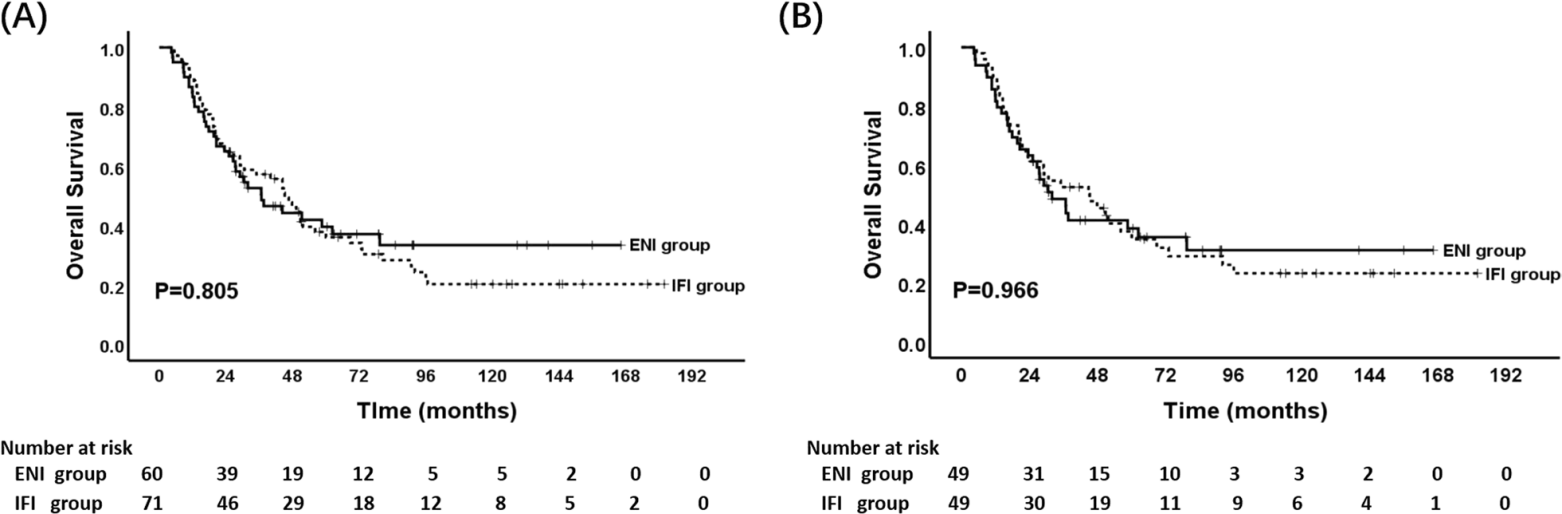
Overall survival for patients in ENI versus IFI group before propensity score-matched analysis (**A**) and after propensity score-matched analysis (**B**). (ENI: elective nodal irradiation; IFI: involved-field irradiation)

At the date of follow-up deadline (October 5, 2022), 7 patients were lost to follow-up. The median follow-up time was 91.1 months (range, 23.8–182.0 months). For the total of 131 patients, the median OS, 1-, 3-, 5-, and 8-year OS rates were 44.4 months, 87.8%, 55.1%, 38.3%, and 27.2%, respectively. Before PSM, the median OS, 1-, 3-, 5-, and 8-year OS rates for ENI and IFI group were 36.8 months, 85.0%, 52.6%, 39.6% and 33.4% versus 45.2 months, 90.1%, 57.3%, 37.8%, 24.3%, respectively. There was no statistical difference between the two groups (*P* = 0.805; HR 1.06, 95% CI 0.69–1.62) (Fig. 2A). After PSM, the median OS, 1-, 3-, 5-, and 8-year OS rates for ENI and IFI group were 32.0 months, 83.7%, 48.5%, 38.5% and 31.1% versus 45.2 months, 89.8%, 52.5%, 37.5%, 26.1% (*P* = 0.966; HR 0.99, 95% CI 0.61–1.61). (Fig. 2B).

On univariate analysis, cN stage (*P* = 0.028; HR 1.21, 95% CI 1.04–1.42) and sex (*P* = 0.039; HR 0.60, 95% CI 0.36–0.99) were shown to be related to OS after PSM (Table 2). And multivariate analysis suggested that N0 stage (*P* = 0.029; HR 1.20, 95% CI 1.02–1.42) and female (*P* = 0.027; HR 0.56, 95% CI 0.34–0.94) were the independent favorable prognostic factors of OS after PSM. A subgroup analysis of OS showed that no benefit was observed from ENI in the comparison with IFI (Table 3, Additional file 1: Fig.A.1–5).

**Table 2:** Univariate analysis of various potential prognostic factors associated with OS

Factor	MST (months)	Survival rate (%)				HR (95% CI)	*P*
		1-y	3-y	5-y	8-y		
Sex							0.039
Male	29.1	84.3	41.9	29.2	17.9	0.60 (0.36–0.99)	
Female	50.8	91.5	62.0	45.8	41.7		
Age at diagnosis (year)							0.427
≤ 65	36.8	86.6	50.7	40.6	29.6	0.81 (0.48–1.37)	
> 65	35.0	87.1	49.4	28.3	21.2		
ECOG PS							0.287
0	36.8	87.2	52.2	39.1	28.7	0.65 (0.29–1.45)	
1–2	23.5	83.3	50.0	–	–		
cT stage							0.966
T3	36.8	86.6	51.9	38.2	26.5	0.99 (0.50–1.94)	
T4	22.7	87.5	42.9	35.7	35.7		
cN stage							0.028
N0	44.8	95.7	51.5	31.6	31.6	1.21 (1.04–1.42)	
N1	60.1	87.5	59.9	50.7	30.1		
N2–3	20.2	77.8	32.9	19.8	19.8		
8th AJCC stage							0.966
II–III	36.8	86.6	51.9	38.2	26.5	0.99 (0.50–1.94)	
IV	22.7	87.5	42.9	35.7	35.7		
Radiation dose							0.353
≥ 50 Gy, ≤ 59.4 Gy	35.0	83.3	47.2	29.1	19.4	1.29 (0.76–2.19)	
> 59.4 Gy, ≤ 66 Gy	36.8	88.2	51.3	40.1	29.9		

**Table 3:** Subgroup analysis of OS by different factors

Characteristics	1-year OS rate (%)		3-year OS rate (%)		5-year OS rate (%)		8-year OS rate (%)		*P*
	ENI	IFI	ENI	IFI	ENI	IFI	ENI	IFI	
Sex									
Male	76.9	92.0	38.1	46.2	32.6	26.9	27.2	10.8	0.858
Female	91.3	87.5	60.6	58.3	45.4	47.7	34.1	41.8	0.784
Age at diagnosis (year)									
≤65	85.7	87.5	48.3	53.1	38.6	42.1	30.1	29.8	0.861
> 65	78.6	94.1	50.0	50.3	40.0	25.2	40.0	16.8	0.950
ECOG PS									
0	86.4	88.1	49.8	54.8	39.5	39.1	31.9	27.2	0.981
1–2	60.0	100.0	–	42.9	–	–	–	–	0.979
cT stage									
T3	85.4	87.8	48.0	55.8	39.0	38.1	29.3	25.4	0.892
T4	75.0	100.0	50.0	33.3	37.5	33.3	–	33.3	0.818
cN stage									
N0	91.7	100.0	48.6	54.5	38.9	24.2	38.9	24.2	0.601
N+	81.1	86.8	48.5	51.9	38.8	41.9	27.6	26.9	0.722
8th AJCC stage									
II–III	85.4	87.8	48.0	55.8	39.0	38.1	29.3	25.4	0.892
IV	75.0	100.0	50.0	33.3	37.5	33.3	–	33.3	0.818
Radiation dose (Gy)									
≥ 50 Gy, ≤ 59.4 Gy	80.0	86.7	58.3	35.6	36.5	23.7	–	23.7	0.551
> 59.4 Gy, ≤ 66 Gy	85.3	91.2	43.7	58.8	37.5	42.9	33.7	28.8	0.637

### Patterns of failure

At the last follow-up visit, a total of 51 patients had treatment failure. The patterns of first failure are shown in Table 4. The incidence of local failure, regional failure and distant failure was 22.4% versus 26.5% (*P* = 0.815), 14.3% versus 4.1% (*P* = 0.159) and 18.4% versus 22.4% (*P* = 0.803) respectively in ENI group and IFI group. For the regional failure pattern, six out of seven patients experienced out-of-field nodal recurrence and one patient was in-filed recurrence in ENI group. Two patients occurred out-of-field nodal recurrence in IFI group. Among the 18 patients with local failure or regional failure in ENI group, 14 (77.8%) underwent salvage radiotherapy and chemotherapy, 2 (9%) chemotherapy, and 2 (9%) best supportive care only. Among the 15 patients with local failure or regional failure in IFI group, 13 (86.7%) underwent salvage radiotherapy and chemotherapy, 2 (13.3%) underwent chemotherapy.

**Table 4:** Patterns of first failure

	ENI (n = 49)		IFI (n = 49)	
	No.	%	No.	%
Local failure	11	22.4	13	26.5
Regional failure	7	14.3	2	4.1
Distant metastasis	9	18.4	11	22.4

### Acute treatment-related toxicities

Among 98 patients, the most common acute toxicities were in grade 1 and 2. None experienced grade 5 acute toxicity. There were 32 patients (65.3%) with ≥ grade 2 acute radiation esophagitis in ENI group and 28 patients (57.1%) in IFI group respectively. Eight patients (16.3%) in ENI group had ≥ grade 2 upper gastrointestinal reaction and five (10.2%) in IFI group. Twenty-nine patients (59.2%) experienced any grade leukocytopenia in ENI group and nineteen patients (38.8%) in IFI group (59.2% vs. 38.8%; *P* = 0.068). Twenty patients (40.8%) in ENI group and 12 patients (24.5%) in IFI experienced ≥ grade 2 leukocytopenia (40.8% vs. 24.5%; *P* = 0.131). Fifteen patients (30.6%) had any grade neutropenia in ENI group and seven (14.3%) in IFI group (30.6% vs. 14.3%; *P* = 0.089). Six patients (12.2%) receiving ENI experienced ≥ grade 2 neutropenia and seven (14.3%) in IFI group (12.2% vs. 14.3%; *P* = 1.000). One patient (2.0%) experienced ≥ grade 2 anemia in ENI group and IFI group respectively. Two patients (4.1%) had thrombocytopenia in IFI group (Additional file 1: Table.A.1).

## Discussion

To the best of our knowledge, our retrospective study had the largest sample size to compare the efficacy of ENI versus IFI in dCCRT for CESCC. The long-term survival of 8 years was reported in our study. No differences were found in 1-, 3-, 5-, 8-year OS between the ENI group and IFI group (*P* = 0.966; HR 0.99, 95% CI 0.61–1.61). More patients had leukocytopenia (59.2% vs. 38.8%) and neutropenia (30.6% vs. 14.3%) in ENI group than IFI group, and no statistical difference in G2 or higher toxicities. Similar locoregional control was obtained in both groups.

The RTOG 85 − 01 trial has demonstrated that dCCRT was superior to radiation alone, with a favorable long-term survival for unresectable thoracic esophageal cancer patients (5-year OS: 26%) [13]. Thereafter, dCCRT has been the standard modality for inoperable esophageal cancer patients. In consideration of the location of cervical esophageal cancer and the destructiveness of surgery, dCCRT is usually the first choice. Even so, some surgeons have explored radical resection techniques in this challenging field, including pharyngo-laryngo-esophagectomy (PLE), larynx-preserving limited resection, robot-assisted cervical esophagectomy and so on [14–16]. Surgical treatment still has a great risk of major complications and a high morbidity and mortality rate [17, 18]. Recently, a retrospective study including 347 cervical esophageal cancer patients from the National Cancer Institute’s Surveillance, Epidemiology, and End Results (SEER) database was done to investigate the survival and prognostic factors of different treatment modalities [19]. The study showed that no significant survival benefit was obtained from triple therapy with surgery over double therapy (median OS time: 31 months vs. 20 months; *P* = 0.184). Similar survival outcomes after curative esophagectomy and radiotherapy were obtained in another retrospective study including 500 Chinese cervical esophageal cancer patients [20]. Among them, only 66 (13.2%) patients with squamous cell carcinoma received chemoradiotherapy. In our study, the 5- and 8-year OS rates of the overall population were 38.3%, and 27.2%, respectively. The OS was satisfactory. With respect to organ preservation and better quality of life, chemoradiotherapy was still recommended as the initial treatment for CESCC.

In our study, N stage was a significant prognostic factor for OS. But the subgroup analysis showed that the patients with regional nodal metastasis did not benefit from ENI. It has been shown that ionizing radiation could provoke tumor-specific immune responses and reshape the immunological tumor microenvironment in a favorable way [21]. Irradiation of lymph nodes may affect the immune system. It was reported that ENI could attenuate chemokine expression, restrained immune infiltration and adversely impacted survival [22]. In practice, for patients with lymph node metastasis, the irradiation field usually included the positive nodal area. For patients with no regional lymph node metastasis, it might be necessary to balance potential benefits against potential risks. In our study, local recurrence and distant metastasis remained the major challenges in the treatment of CESCC with dCCRT. For the regional failure pattern, six out of seven patients experienced out-of-field nodal recurrence and one patient was in-filed recurrence in ENI group. Two patients occurred out-of-field nodal recurrence in IFI group (4.1% vs.14.3%, *P* = 0.159). McDowell et al. [23] analyzed the failure patterns of 81 CESCC patients receiving RT or CRT. There were 34 local (42%), and 34 distant (42%) failures respectively. Zhao et al. [24] evaluated the failure patterns of CESCC treated by definitive radiotherapy with or without concurrent chemotherapy. The results showed that eight patients had regional failures, among whom 2 had failures within the CTV and 6 out of CTV. In our study, the 1-, 3-, 5-, and 8-year OS rate of patients with N0 stage receiving IFI was 100.0%, 54.5%, 24.2%, 24.2%, respectively, which was comparable to the patients receiving ENI (Additional file 1: Fig.A.2). Considering chemotherapy might contribute to the control of micrometastasis as a systematic treatment modality, the conclusion probably not be applicable to patients treated with radiotherapy alone.

There are some limitations to this study. It was a retrospective, non-randomized and single-institutional study. Potential confounding factors such as patient social economic status, bodyweight loss, biomarkers, and chemotherapy regimens might affect the final survival. Clinical data of our study was from a long large time span of 14 years, in which selection treatment bias may exist, particularly clinician’s and patient’s preference. Regretfully, the treatment selection bias was difficult to avoid for this retrospective study even if we had carried out propensity score matching. In addition, PET was not available for all patients. Accurate diagnosis before treatment is especially important to determine the location of the primary tumor and lymph node metastasis. Therefore, well-designed, larger multi-center prospective trials are necessary for better insight into the effect of ENI on CESCC.

In conclusion, cervical esophageal squamous cell carcinoma patients undergoing definitive concurrent chemoradiotherapy has a satisfactory prognosis with organ conservation. N + stage and male have worse prognosis. The patients with regional nodal metastasis cannot benefit from ENI in select cases, the involved-field irradiation might be a better alternative in the treatment of CESCC.

### Supplementary Information

**Additional file 1: Fig. A.1.** Overall survival for patients with different clinical T stage (cT) in ENI versus IFI group after propensity score-matched analysis. **Fig. A.2.** Overall survival for patients with different clinical N stage (cN) in ENI versus IFI group after propensity score-matched analysis. **Fig. A.3.** Overall survival for patients with clinical TNM stage (cTNM) in ENI versus IFI group after propensity score-matched analysis. **Fig. A.4.** Overall survival for patients receiving different radiation doses in ENI versus IFI group after propensity score-matched analysis. **Fig. A.5.** Overall survival for patients with hypopharyngeal invasion or not in ENI versus IFI group after propensity score-matched analysis. **Table A.1.** Acute toxicity of patients with CESCC receiving dCCRT.

## Data Availability

All data generated or analyzed during this study are available in this published article and its Additional file 1.

## Abbreviations

dCCRT: Definitive concurrent chemoradiotherapy
CEC: Cervical esophageal cancer
CESCC: Cervical esophageal squamous cell carcinoma
ENI: Elective nodal irradiation
IFI: Involved-field irradiation
PSM: Propensity score matching
OS: Overall survival
ECOG: Eastern Cooperative Oncology Group
3D-CRT: Three-dimensional conformal radiotherapy
IMRT: Intensity-modulated radiotherapy
HR: Hazard ratio
CI: Confidence interval
