# Supplementary material for: Elective nodal irradiation versus involved-field irradiation for stage II–IV cervical esophageal squamous cell carcinoma patients undergoing definitive concurrent chemoradiotherapy: a retrospective propensity study with 8-year survival outcomes
Source: Radiat Oncol. 2023 Aug 28;18:142. doi: 10.1186/s13014-023-02332-2 (PMC10464095; doi:10.1186/s13014-023-02332-2)
Supplement: Supplementary file 1 — Additional file 1: Fig. A.1. Overall survival for patients with different clinical T stage (cT) in ENI versus IFI group after propensity score-matched analysis. Fig. A.2. Overall survival for patients with different clinical N stage (cN) in ENI versus IFI group after propensity score-matched analysis. Fig. A.3. Overall survival for patients with clinical TNM stage (cTNM) in ENI versus IFI group after propensity score-matched analysis. Fig. A.4. Overall survival for patients receiving different radiation doses in ENI versus IFI group after propensity score-matched analysis. Fig. A.5. Overall survival for patients with hypopharyngeal invasion or not in ENI versus IFI group after propensity score-matched analysis. Table A.1. Acute toxicity of patients with CESCC receiving dCCRT. [file 13014_2023_2332_MOESM1_ESM.docx]

Supplemental materials

Fig.A.1 Overall survival for patients with different clinical T stage (cT) in ENI versus IFI group after propensity score-matched analysis.

Fig.A.2 Overall survival for patients with different clinical N stage (cN) in ENI versus IFI group after propensity score-matched analysis.

Fig.A.3 Overall survival for patients with clinical TNM stage (cTNM) in ENI versus IFI group after propensity score-matched analysis.

Fig.A.4 Overall survival for patients receiving different radiation doses in ENI versus IFI group after propensity score-matched analysis.

Fig.A.5 Overall survival for patients with hypopharyngeal invasion or not in ENI versus IFI group after propensity score-matched analysis.

Table A.1 Acute toxicity of patients with CESCC receiving dCCRT.

**Fig.A.1 Overall survival for patients with different clinical T stage (cT) in ENI versus IFI group after propensity score-matched analysis.**


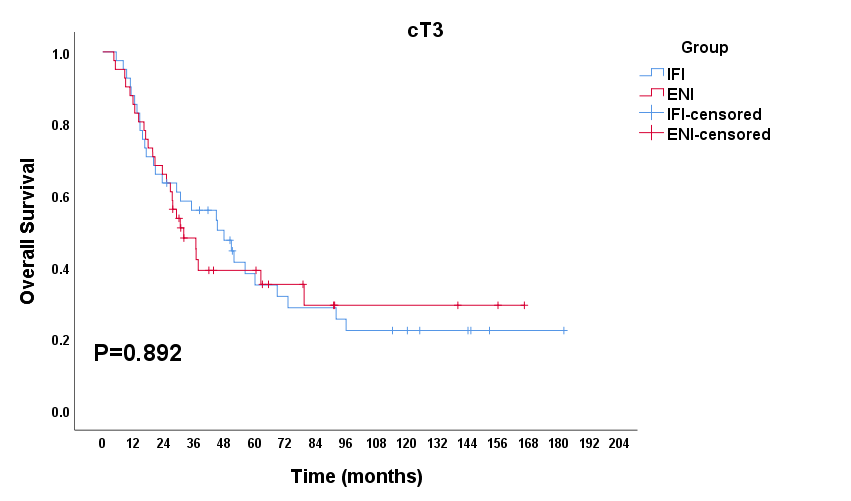


Fig.A.1.1 Overall survival for patients with cT3 in ENI versus IFI group after propensity score-matched analysis.


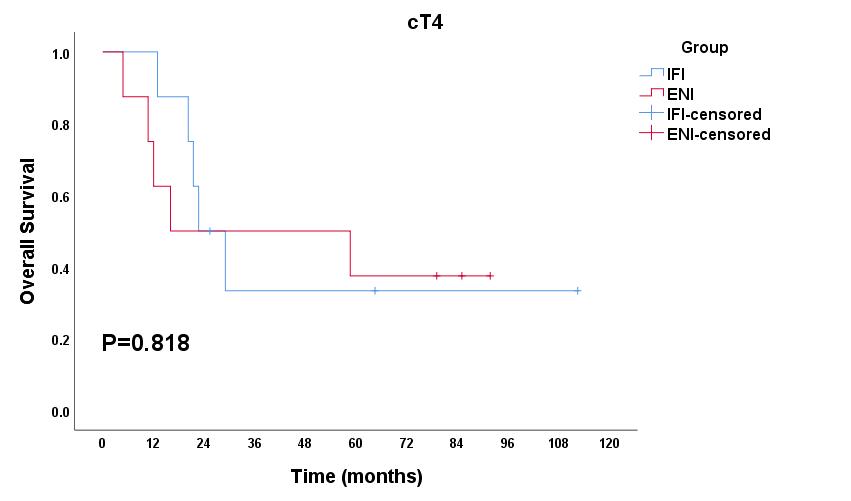


Fig.A.1.2 Overall survival for patients with cT4 in ENI versus IFI group after propensity score-matched analysis.

**Fig.A.2 Overall survival for patients with different clinical N stage (cN) in ENI versus IFI group after propensity score-matched analysis.**


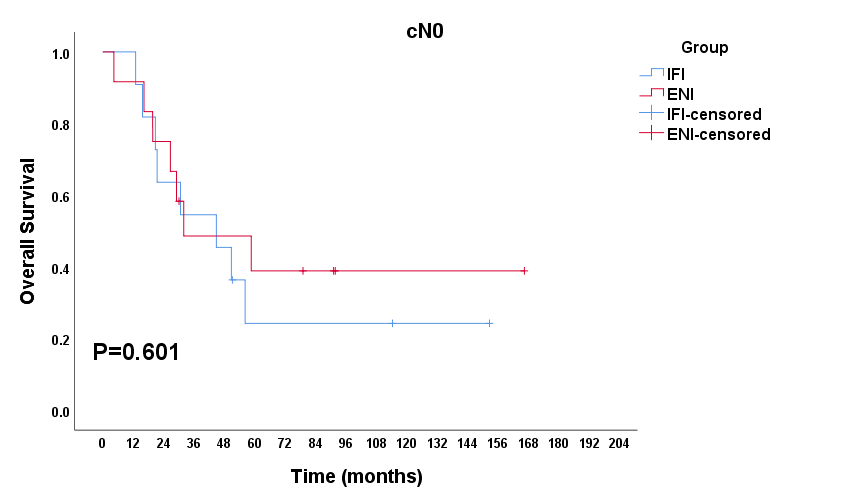


Fig.A.2.1 Overall survival for patients with cN0 in ENI versus IFI group after propensity score-matched analysis.


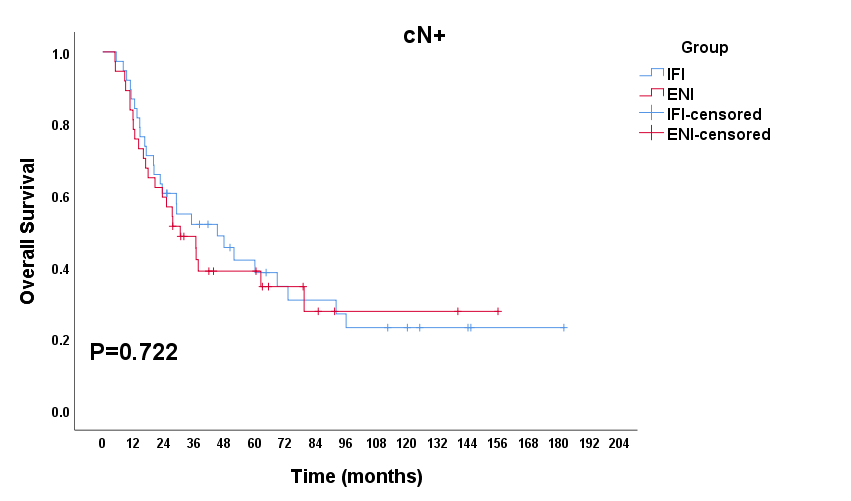


Fig.A.2.2 Overall survival for patients with cN+ in ENI versus IFI group after propensity score-matched analysis.

**Fig.A.3 Overall survival for patients with clinical TNM stage (cTNM) in ENI versus IFI group after propensity score-matched analysis.**


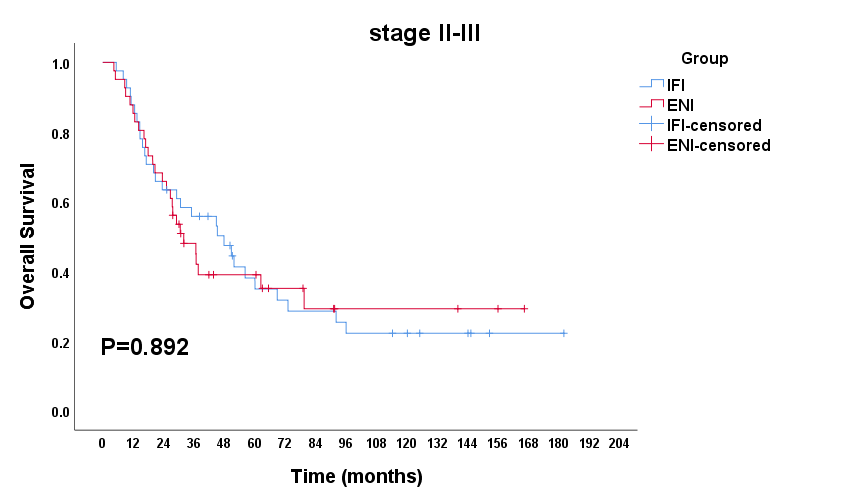


Fig.A.3.1 Overall survival for patients with stage Ⅱ-Ⅲ in ENI versus IFI group after propensity score-matched analysis.


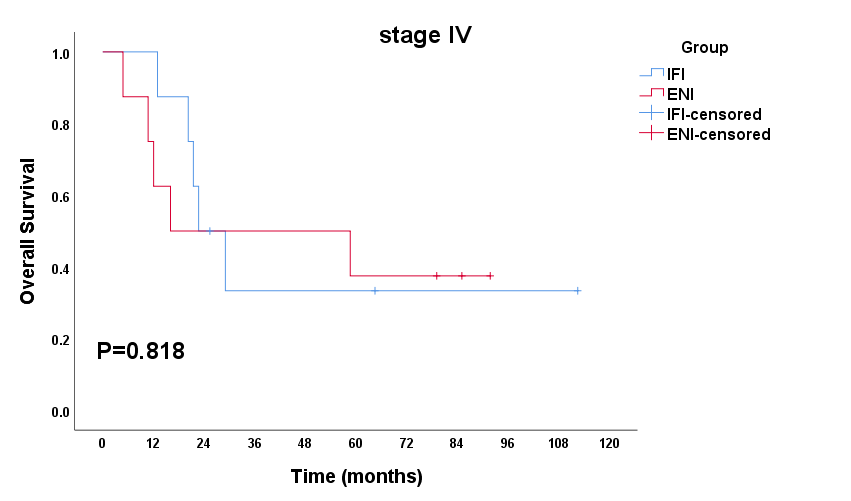


Fig.A.3.2 Overall survival for patients with stage Ⅳ in ENI versus IFI group after propensity score-matched analysis.

Fig.A.4 Overall survival for patients receiving different radiation doses in ENI versus IFI group after propensity score-matched analysis.

**Fig.A.5 Overall survival for patients with hypopharyngeal invasion or not in ENI versus IFI group after propensity score-matched analysis.**


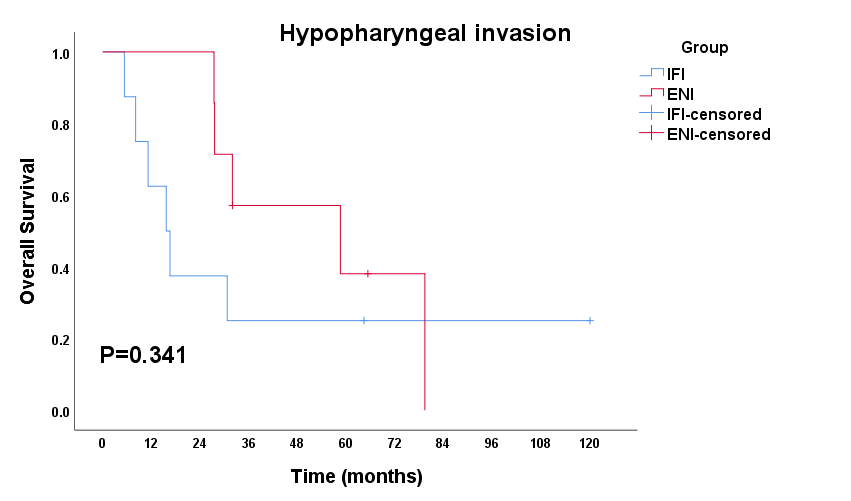


Fig.A.5.1 Overall survival for patients with hypopharyngeal invasion in ENI versus IFI group after propensity score-matched analysis.


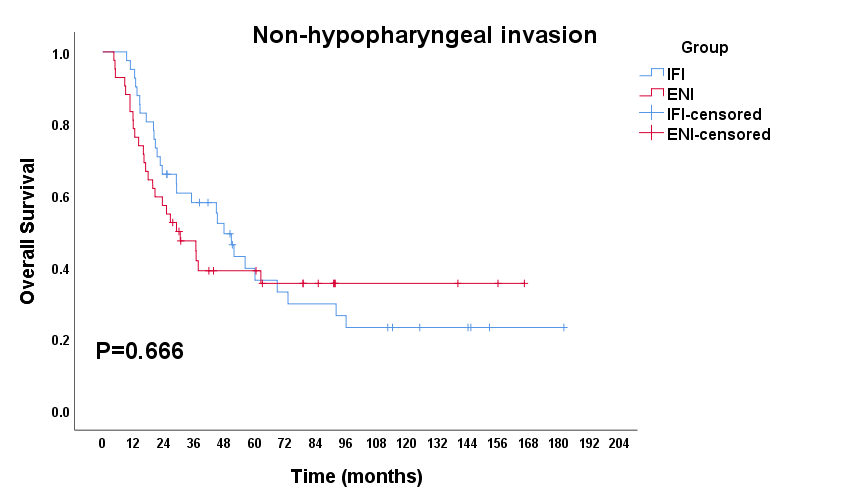


Fig.A.5.2 Overall survival for patients without hypopharyngeal invasion in ENI versus IFI group after propensity score-matched analysis.

Table A.1 Acute toxicity of patients with CESCC receiving dCCRT.

| Toxicity | Group | Classification | | | | |
| --- | --- | --- | --- | --- | --- | --- |
|  |  | Grade 0  n (%) | Grade 1  n (%) | Grade 2  n (%) | Grade 3  n (%) | Grade 4  n (%) |
| Acute radiation esophagitis | ENI | 8 (16.3) | 9 (18.4) | 30 (61.2) | 1 (2.0) | 1 (2.0) |
|  | IFI | 7 (14.3) | 14 (28.6) | 28 (57.1) | 0 (0.0) | 0 (0.0) |
| Acute radiation pneumonitis | ENI | 46 (93.9) | 0 (0.0) | 2 (4.1) | 1 (2.0) | 0 (0.0) |
|  | IFI | 47 (95.9) | 2 (4.1) | 0 (0.0) | 0 (0.0) | 0 (0.0) |
| Upper gastrointestinal reaction | ENI | 36 (73.5) | 5 (10.2) | 6 (12.2) | 2 (4.1) | 0 (0.0) |
|  | IFI | 40 (81.6) | 4 (8.2) | 5 (10.2) | 0 (0.0) | 0 (0.0) |
| Leukocytopenia | ENI | 20 (40.8) | 9 (18.4) | 15 (30.6) | 4 (8.2) | 1 (2.0) |
|  | IFI | 30 (61.2) | 7 (14.3) | 8 (16.3) | 4 (8.2) | 0 (0.0) |
| Neutropenia | ENI | 34 (69.4) | 9 (18.4) | 2 (4.1) | 3 (6.1) | 1 (2.0) |
|  | IFI | 42 (85.7) | 0 (0.0) | 5 (10.2) | 2 (4.1) | 0 (0.0) |
| Lymphocytopenia | ENI | 3 (6.1) | 3 (6.1) | 18 (36.7) | 24 (49.0) | 1 (2.0) |
|  | IFI | 1 (2.0) | 8 (16.3) | 12 (24.5) | 24 (49.0) | 4 (8.2) |
| Anemia | ENI | 34 (69.4) | 14 (28.6) | 1 (2.0) | 0 (0.0) | 0 (0.0) |
|  | IFI | 33 (67.4) | 15 (30.6) | 1 (2.0) | 0 (0.0) | 0 (0.0) |
| Thrombocytopenia | ENI | 49 (100.0) | 0 (0.0) | 0 (0.0) | 0 (0.0) | 0 (0.0) |
|  | IFI | 47 (95.9) | 1 (2.0) | 1 (2.0) | 0 (0.0) | 0 (0.0) |
